# Review of the Nearctic genus *Lacconotus* LeConte (Coleoptera, Mycteridae, Eurypinae)

**DOI:** 10.3897/zookeys.162.1998

**Published:** 2012-01-05

**Authors:** Darren Pollock, Christopher G. Majka

**Affiliations:** 1 Department of Biology, Eastern New Mexico University, Portales, New Mexico, USA; 2 Research Associate, Nova Scotia Museum, 1747 Summer Street, Halifax, Nova Scotia, Canada

**Keywords:** Coleoptera, Mycteridae, Eurypinae, North America, new records, range extensions

## Abstract

*Lacconotus* LeConte, the sole Nearctic representative of the eurypine Mycteridae, is revised, based on morphological features of adults. The following **syn. n.** is proposed: *Lacconotus pallidus* Van Dyke, 1928 = *Lacconotus pinicola* Horn, 1879. The former is a light-colored form with a southern California distribution. A **subgen. n.,** *Alcconotus*, is described for *Lacconotus pinicola*, producing the following **comb. n.**: *Lacconotus (Alcconotus) pinicola* (Horn). A lectotype is designated for *Lacconotus pinicola*. A key separating the two subgenera and species is provided, as are photographs and illustrations of salient structures of adults, and maps showing collection localities. *Lacconotus punctatus* is newly recorded in Alabama, Arkansas, Massachusetts, Oklahoma, Texas, and Wisconsin; *Lacconotus pinicola* is newly recorded in Arizona and Utah in the USA, and Baja California Norte in Mexico. Phenology information shows a north-to-south gradation in occurrence time.

## Introduction

Among the three subfamilies of Mycteridae, the Eurypinae (= Lacconotinae) are the most diverse with 26 genera and 160 species recognized worldwide, the greatest diversity being in New and Old World tropical regions (Pollock 2010). The Nearctic fauna of Mycteridae is not large, but represents all three subfamilies: six species of *Mycterus* Clairville (Mycterinae), three species of *Hemipeplus* Latreille (Hemipeplinae), and two species of Eurypinae. The latter are represented by *Lacconotus punctatus* LeConte and *Lacconotus pinicola* Horn (= *Lacconotus pallidus* Van Dyke). The distribution of the family in North America is decidedly either western or eastern, i.e., there are no species represented in the interior of the continent, and no single species bridges this distributional gap (Pollock 2002).

According to Horn (1879: 339), “The Mycteridae seem to have been cast about from place to place by the various students who have had occasion to write about them.” Indeed, the constituents of the ‘modern’ concept of Mycteridae have been placed in multiple families, ranging from Cucujidae (for *Hemipeplus*) (e.g., LeConte 1854), to Melandryidae (e.g., LeConte and Horn 1883; Van Dyke 1928), and Pythidae (e.g., Seidlitz 1917; Blair 1928). In a phylogenetically based analysis of families related to Mycteridae, Beutel and Friedrich (2005) elucidated the following relationship: (Prostomidae + (Mycteridae + Boridae)); however, they stated that the relationships among the Mycteridae and related families are far from settled.

This study of *Lacconotus* was undertaken for several reasons. For some time, it was recognized (Pollock, personal observation) that the eastern and western species of the genus were rather dissimilar structurally; recent collections of specimens, especially through Michael Caterino’s “California Beetle Project”, have added much more material for study; and, it is the first author’s goal to revise all genera of world Mycteridae, including the many presently poorly known genera of Eurypinae.

## Natural History

As with many other groups of Tenebrionoidea, and Coleoptera generally, relatively little is known of the specific habits of eurypine Mycteridae. Larvae have been described for only a few species; these descriptions (see references in Pollock 2010) indicate that larvae occur under loosened tree bark (e.g., *Physcius fasciatus* Pic, *Physiomorphus* spp., *Phaeogala rufa* Abdullah) or in palm leaf axils or dead foliage (e.g. *Eurypus* spp.). With the exception of *Stilpnonotus* spp., eurypine larvae have flattened bodies and well-developed, complex urogomphal plates, typical of larvae that move subcortically. Lawrence (1991) indicated that mycterid larvae are phytophagous, and that plant-derived material has been found in gut contents of several species.

The biology of *Lacconotus* seems typical of eurypines: larvae occur under bark of various tree species. Lawrence (1991) illustrated the larva of *Lacconotus pinicola* (Apache Co., Arizona) and indicated that larvae of this species occur under bark of poplar (*Populu*s) and fir (*Abies*). Crowson and de Viedma (1964) mentioned a larva, presumed to be that of *Lacconotus pallidus* (= *Lacconotus pinicola*), from under bark of dead oak (*Quercus* sp.).

Other specific details pertinent to natural history, derived from label data or other sources, are given under *Lacconotus punctatus* and *Lacconotus pinicola*, below.

## Methods and conventions

Standard taxonomic methods were used in this study. Habitus photographs were taken with a Nikon Coolpix 5000^®^ digital camera fitted to a Leica MZ95 stereoscope. Approximately 30 separate photographs were taken for each specimen/structure; these were imported into Combine ZP (Hadley 2010), which stacked and aligned the individual images to create a final photograph completely in focus.

Several measurements were used: HL = length of head from anterior margin of pronotum to labrum; PL = length of pronotum along middle; EL = length of elytron from anterior to posterior extent; GHW = maximum width of head, across eyes; GPW = maximum pronotal width; GEW = maximum width of both elytra; TL = HL + PL + EL.

Label data on type specimens are recorded *verbatim*, with all label data enclosed in quotes and individual labels separated by a slash (/). Information added by the authors for clarity is enclosed in square brackets ([ ]).

Abbreviations of collections (largely following Evenhuis 2011) consulted and referred to in this study are:

AAAC Albert A. Allen Collection, Boise, Idaho, USA

AMNH American Museum of Natural History, New York City, New York, USA

CAS California Academy of Sciences, San Francisco, California, USA

CMNH Carnegie Museum of Natural History, Pittsburgh, Pennsylvania, USA

CNC Canadian National Collection of Insects, Arachnids, and Nematodes, Ottawa, Ontario, Canada

CSCA California State Collection of Arthropods, Sacramento California, USA

CUIC Cornell University Insect Collection, Ithaca, New York, USA

DAPC Darren A. Pollock Collection, Eastern New Mexico University, Portales, New Mexico, USA

EMEC Essig Museum of Entomology, University of California, Berkeley, California, USA

FMNH Field Museum of Natural History, Chicago, Illinois, USA

MCZ Museum of Comparative Zoology, Harvard University, Cambridge, USA

NMNH National Museum of Natural History, Washington, District of Columbia, USA

NSMC Nova Scotia Museum, Halifax, Nova Scotia, Canada

QMOR Collection Entomologique Ouellet-Robert, Université de Montréal, Montréal, Québec, Canada

RBC Rick Buss Collection, Albuquerque, New Mexico, USA

SBMN Santa Barbara Museum of Natural History, Santa Barbara, California, USA

TAMU Texas A & M University, College Station, Texas, USA

UAIC University of Arizona Insect Collection, Tucson, Arizona, USA

UBC University of British Columbia, Vancouver, British Columbia, Canada

UCR University of California-Riverside, Riverside, California, USA

UNHC University of New Hampshire, Durham, New Hampshire, USA

WFBM W.F. Barr Entomological Collection, University of Idaho, Moscow, Idaho, USA

WIRC WisconsinInsect Research Collection, University of Wisconsin, Madison, Wisconsin, USA

## Identification

Adults of *Lacconotus* can be differentiated with the following key:

**Table:** 

1	Dorsal color dark brown to near black, except for variously developed lateral reddish areas on pronotum (Fig. 1) (in some specimens reduced to the extreme posterolateral corners, e.g., Fig. 4); elytral punctation coarse, punctures obvious; antennae short, antennomeres submoniliform; male sex patch large, bulbous, glabrous, and with contrasting pink-red color (Fig. 5); known from eastern North America, west to Texas (Fig. 15)	*Lacconotus (Lacconotus) punctatus* LeConte
–	Dorsal color uniform, light brown (Fig. 3) to nearly black (Fig. 2); punctation of elytra fine, punctures not conspicuous; antennae relatively long, antennomeres longer than wide; male sex patch small, oval, setose, and not distinctly contrasting in color to venter (Fig. 6); known from western North America, east to Colorado and New Mexico (Fig. 15)	*Lacconotus (Alcconotus) pinicola* Horn

### Lacconotus

LeConte

http://species-id.net/wiki/Lacconotus

Lacconotus LeConte 1862: 255. – Gemminger and Harold 1870: 2179; Horn 1879: 338; Austin 1880: 41; LeConte and Horn 1883: 401; Fall 1901: 177; Dury 1902: 174; Blatchley 1910: 1302; Seidlitz 1917: 99; Leng 1920: 240; Leng and Mutchler 1933: 25, 36; Blair 1928: 33; Spilman 1951: 48; Spilman 1952: 10-11; Spilman 1954: 89; Arnett 1963: 717; Hatch 1965: 88; Campbell 1991: 267; Lawrence and Newton 1995: 896; Poole and Gentili 1996: 315; Arnett 2000: 473; Pollock 2002: 532; Bouchard et al. 2011: 443. **Type species:***Lacconotus punctatus* LeConte, by monotypy.

#### Description.

[note: “*Lacconotus*” indicates the character states for subgenus *Lacconotus*, while “*Alcconotus*” refers to the corresponding states in subgenus *Alcconotus* (see below)].

Body elongate oval (TL/GEW 2.8-3.4), parallel-sided to widened posterior of middle, slightly (*Alcconotus*) to moderately (*Lacconotus*) flattened dorsally. TL 4.6–7.5 mm.

Head relatively short, narrowed slightly posterior of eyes; eyes moderately large, distinctly convex, inner margins convergent anteriorly; facets moderately coarse, with intrafacetal setae (especially noticeable in *Alcconotus*); frontoclypeal suture indistinct, indicated by slight furrow only; antennal insertions slightly concealed dorsally by raised lateral margins of frons; labrum transverse, anterior margin straight to shallowly sinuate.

**Figure 1.**
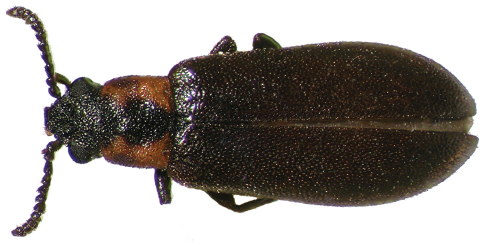
Dorsal habitus photograph of *Lacconotus (Lacconotus) punctatus*; female, New Hampshire. TL = 5.1 mm. Photo credit: Darren Pollock, Eastern New Mexico University.

**Figure 2.**
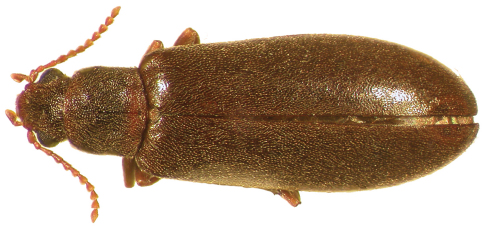
Dorsal habitus photograph of *Lacconotus (Alcconotus) pinicola*; female, Utah. TL = 6.3 mm.<br/> Photo credit: Darren Pollock, Eastern New Mexico University.

**Figure 3.**
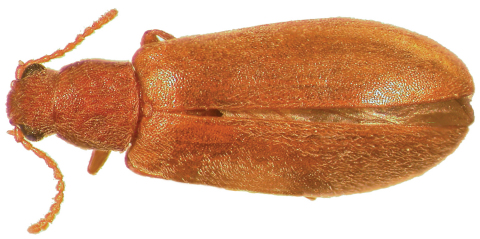
Dorsal habitus photograph of *Lacconotus (Alcconotus) pinicola* (‘*pallidus*'); female, California. TL = 6.5 mm. Photo credit: Darren Pollock, Eastern New Mexico University.

Antennae relatively short (Fig. 1) (*Lacconotus*) to moderately elongate (Figs 2–3) (*Alcconotus*), not exhibiting distinct sexual dimorphism; scape and pedicel moniliform (*Lacconotus*) to slightly elongate (*Alcconotus*); antennomere 3 elongate, antennomeres 4–10 wider than long, submoniliform (*Lacconotus*) to elongate, triangular to subserrate (*Alcconotus*); antennomere 11 narrowed distally; sensilla present on distal surface of antennomeres 5–10.

Mandibles relatively short, stout, slightly asymmetrical, apically bidentate; terebral teeth absent, or represented by several minute crenulae; molae approximately equal in size, subquadrate, with slightly developed surface texture; both mandibles with abrupt incision distal of mola; ventral row of microtrichia absent; prostheca distinct, about half length of mandible, inserted near distal edge of mola; maxilla with galea slightly longer than lacinia; galea bluntly rounded distally, relatively densely pubescent; maxillary palpi elongate, apical palpomere securiform (*Lacconotus*) to slightly cultriform (*Alcconotus*); inner margins of palpomeres 1 and 2 straight (*Lacconotus*) to slightly sinuate (*Alcconotus*); mentum short, about 2 × wider than long, posterior suture straight (*Alcconotus*) or distinctly arcuate (*Lacconotus*).

Thorax. Prothorax subquadrate (Figs 1–3), slightly wider than long (GPW/PL = 0.90–1.29); pronotal margins straight and convergent anteriorly, to slightly arcuate and widest near midlength; pronotal disc flat to slightly convex, with variously developed shallow, paired depressions; lateral pronotal carinae absent, margin smooth; posterior margin with pair of small, deep, punctiform pits; prosternum anterior of procoxae short (*Lacconotus*) to moderately elongate (*Alcconotus*), surface flat to slightly sunken medially; intercoxal process short, knife-like, extended to about half length of procoxae; procoxae rounded (*Lacconotus*) to elongate (*Alcconotus*); procoxal cavities open externally and internally; protrochantin concealed.

Elytra elongate, subovate, disc flat (*Lacconotus*) to slightly convex (*Alcconotus*), upper surface uniformly and moderately coarsely punctate and setose (slightly more coarse in *Lacconotus*), setae closely appressed to elytral surface; apical elytral patch present, but not conspicuous dorsally, not contrasting in color with respect to remainder of elytron; epipleuron narrow, traceable only to abdominal ventrite 3 or 4; mesosternum with posterior intercoxal process parallel-sided, extended posteriorly to near posterior extent of mesocoxae; mesocoxae narrowly but completely separated, trochantins exposed; coxal cavities partly closed laterally by mesepimera; metasternum large, convex, anterior margin with indistinct (*Lacconotus*) to distinct (*Alcconotus*) process, in contact with posterior mesosternal process; median impressed line distinct to at least half distance to anterior margin of metasternum; metendosternite with long, relatively wide stalk; anterior tendons inserted on anterior margin of metendosternite body; laminae large, produced and somewhat angulate laterally.

Wing (Figs 7–8) fully developed, membrane beyond distinct radial cell moderately short (esp. in *Lacconotus*); venation similar in both species, but wing membrane and veins relatively darkly pigmented in *Alcconotus* (Fig. 8), very pale in *Lacconotus* (Fig. 7); wedge cell present; 3 MP veins reaching wing margin, proximal to CuA+AA; pigmented patches (flecks) present near junction of RP and MP, and near radial cell (*Alcconotus*), indistinct in *Lacconotus*.

Legs well developed, similar in relative shape and size on all thoracic segments; middle and hind femora slightly more expanded than front femora; femora relatively slender, but distinctly widened toward midlength; tibiae straight, about same length as femora, tibial spurs very short, equal in length; tarsomeres slender, 5–5-4; all tarsomeres simple ventrally, except for penultimate tarsomere with large ventral lobe; basal tarsomere on hind tarsus subequal in length to other tarsomeres combined; tarsal claws with large blunt tooth.

Abdomen with all ventrites freely articulated, uniformly punctate and setose, except for male sex patch; sex patch of two forms: small, longitudinally oval, setose patch on ventrite 2, not contrasting in color with ventrite (Fig. 6) (*Alcconotus*); or large, somewhat bulbous, glabrous area occupying and somewhat distorting the shape of ventrite 2, distinctly contrasting in color to dark ventrite surface (Fig. 5) (*Lacconotus*).

Male genitalia with median lobe dorsal to tegmen; sternite 9 forming ring-like sclerite, U-shaped in *Alcconotus* (Fig. 11), Y-shaped in *Lacconotus* (Fig. 9); tegmen relatively short, stout; basale broader than long, proximal margin deeply emarginate; length of apicale subequal to that of basale (along lateral margins); parameres of apicale short (Fig. 11) (*Alcconotus*) to slightly elongated (Fig. 9) (*Lacconotus*), with distal, inwardly-directed hook; median lobe (Figs 10, 12) stout, longer than tegmen; ventral side deeply emarginate, dorsal side proximally subquadrate, laterally produced, explanate; apex of median lobe triangular, relatively blunt.

Female genitalia with elongate, flexible, and only slightly sclerotized ovipositor; coxites 4-segmented, sparsely setose; distal segment short, distinctly more sclerotized than remainder of coxite; styli short, setose, with several very long distal setae; dorsal and ventral baculi well developed, extended to base of coxites; spiculum long, far exceeding length of segment 8; bursa copulatrix small (Fig. 13) (*Lacconotus*) to very large (Fig. 14) (*Alcconotus*), separated from vagina by narrow constriction, without conspicuous surface texture; spermatheca present, inserted near or at base of bursa, with elongate spermathecal gland.

### Lacconotus (Lacconotus) punctatus

LeConte

http://species-id.net/wiki/Lacconotus_punctatus

Figs 1 4 –5 7 9–10 13 15 –16

Lacconotus punctatus LeConte 1862: 255. –Type locality: “Pennsylvania.” Gemminger and Harold 1870: 2179; Dury 1902: 174; Blatchley 1910: 1302; Seidlitz 1917: 99; Leng 1920: 240; Blair 1928: 33; Van Dyke 1928: 257; Spilman 1954: 89, 93; Arnett 1983: 3; Campbell 1991: 267; Poole and Gentili 1996: 315; Pollock 2002: 530 (fig. 9.112), 532; Majka 2006: 38; Majka and Selig 2006; Ulyshen et al. 2010.

#### Type.

 holotype, male, labeled: “[pink circle] / ♂ / Type 4760 / Lacconotus punctatus Lec. / HOLOTYPE ♂ Lacconotus punctatus LeC. exam. Pollock 2000”, in MCZC.

#### Diagnosis.

**Figure 4.**
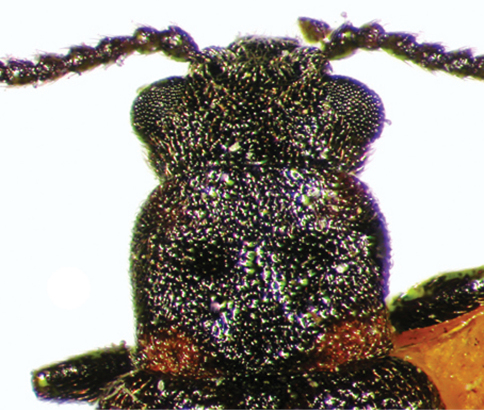
Forebody of *Lacconotus (Lacconotus) punctatus*, dark form. Photo credit: Darren Pollock, Eastern New Mexico University.

**Figures 5–6.**
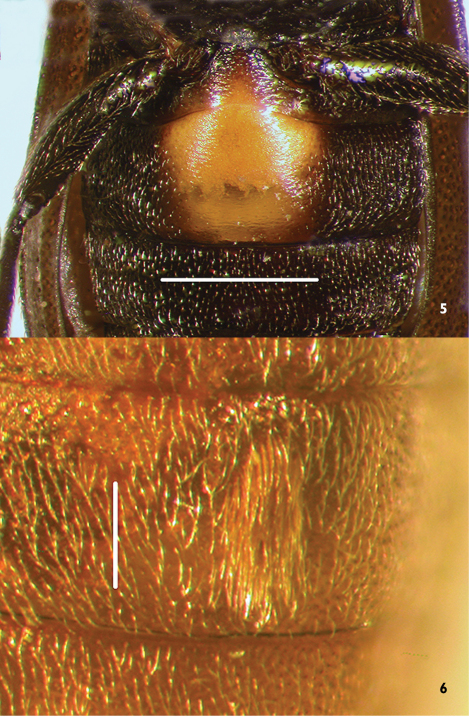
Male sex patch of species of *Lacconotus* **5** *Lacconotus (Lacconotus) punctatus*, scale bar = 0.75 mm; **6** *Lacconotus (Alcconotus) pinicola*, scale bar = 0.25 mm. Photo credit: Darren Pollock, Eastern New Mexico University.

 This species is easily diagnosed by the following characteristics: body color dark piceous to near black, pronotum with reddish margins and black center (Figs 1, 4); antennae relatively short, antennomeres submoniliform; male sex patch on ventrite 2 bulging, glabrous, yellow-orange, contrasting with dark color of venter (Fig. 5); distribution in eastern North America (Fig. 15).

#### Re-description.

 To general features of *Lacconotus* (see description, above) the following can be added: TL 4.4–5.8 mm; GEW 1.5–2.0 mm; TL/GEW 2.9–3.3. Dorsal body surface uniformly piceous to near black, except for lateral areas of pronotum red-orange (Fig. 1); extent of light area varying, from extreme posterolateral corners to fully 2/3 of pronotal disc; ventral surface and legs dark, piceous to near black; antennomeres 5–10 short, distinctly wider than long, submoniliform; antennal sensilla completely annular, covering entire distal antennal surface, around insertion point of next antennomere; wing very pale, veins present, but inconspicuous; male sex patch (Fig. 5) very large, occupying entire length of second ventrite, prolonged onto ventrite one, glabrous and bulging ventrally; color of sex patch yellow-orange, distinctly contrasting background color of ventrite; tegmen of male genitalia (Fig. 9) moderately elongate, parameres of apicale relatively slender; bursa copulatrix (Fig. 13) spherical, small.

*Lacconotus punctatus* is newly recorded in Alabama, Arkansas, Massachusetts, Oklahoma, Texas, and Wisconsin (see Appendix A). Published records of *Lacconotus punctatus* are from Ontario (Campbell 1991)^1^, Québec (Campbell 1991)^2^, and Nova Scotia (Majka and Selig 2006) in Canada [Horn (1879) first reported it from “Canada”], and Georgia (Ulyshen et al. 2010), Michigan (Hubbard et al. 1878; Horn 1879), New Hampshire (Chandler 2001), Ohio (Dury 1902; Blatchley 1910), and Pennsylvania (LeConte 1862) in the United States.
Although listed from Ontario in Campbell (1991), we have not been able to find any published record, or any vouchers specimen in any North American collection that would substantiate this report. Consequently, pending verification of its occurrence in this jurisdiction, we remove Ontario from the known distribution of this species.
In addition to the specimen from Montreal in the CUIC (Appendix 1) a second specimen from Québec was formerly in the Ouellet-Robert collection of the Université de Montréal, however, the specimen was stolen before being databased so its collection date and locality in the province are unknown (pers. com., Louise Cloutier)

The new records above make clear that the distribution of *Lacconotus punctatus* in North America is much wider than previously known (Fig. 15). Less than a decade ago, Pollock (2002) reported the species from only four jurisdictions in North America (Québec, Pennsylvania, Ohio, and Michigan). There are now records from 14 states and provinces on the continent. The records from Wisconsin constitute a northwestern range extension of 650 km; and those from Oklahoma a southwestern range extension of 1,000 km. The present distribution indicates that *Lacconotus punctatus* is found over much of eastern North America, from a latitude of 33.6° to 44.3°N, and between longitudes of 64.5° and 95.3°W, much of the continent west of the prairies.

There is also much more information on the range of habitats that *Lacconotus punctatus* occupies. In Nova Scotia a specimen was found in a mixed forest of white pine (*Pinus strobus* L.), balsam fir (*Abies balsamea* (L.) Mill), eastern hemlock (*Tsuga canadensis* (L.) Carr.), and maple (*Acer* spp.) (Majka and Selig 2006). In New Hampshire, W.J. Morse and D.S. Chandler collected 26 specimens at a water tower in a mixed hardwood forest with eastern hemlocks. In Oklahoma a specimen was collected on a dead oak and in Wisconsin a specimen was found in an oak savanna. In Arkansas a specimen was found in a mixed forest/old field.

In Georgia, specimens were collected in mature bottomland hardwood forests in April with flight intercept traps in the forest canopy (Ulyshen et al. 2010). Dominant trees included box elder (*Acer negundo* L.), oak (*Quercus* spp.), ash (*Fraxinus* spp.), eastern cottonwood (*Populus deltoides* (Bartr.) ex. Marsh.), and sweetgum (*Liquidambar styraciflua* L.) with some loblolly pine (*Pinus taeda*) (M. Ulyshen, pers. comm.). Five of six specimens were found 15 m above the forest floor (Ulyshen et al. 2010). Ulyshen et al. (2010) proposed that *Lacconotus punctatus* may be an early-seasonal canopy specialist, a reason why it has been so infrequently collected.

The phenology information that is available (Fig. 16) indicates that adults can be found between 31 March and 16 June. Specimens from southern areas (i.e., Georgia) were found in mid April (Ulyshen et al. 2010), whereas those from northern latitudes (i.e., New Hampshire) occur mainly during the last two weeks of May and first week of June (D.S. Chandler, pers. comm.), indicating a north-to-south gradation in occurrence period.

**Figures 7–8.**
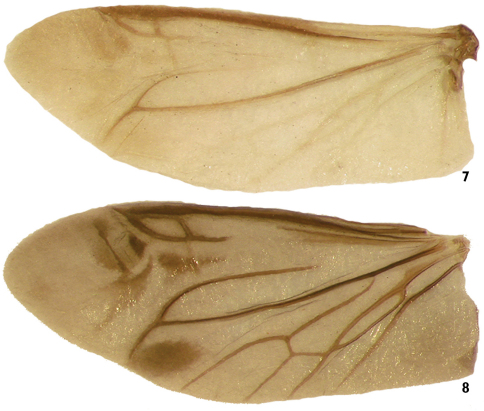
Wing of species of *Lacconotus* **7** *Lacconotus (Lacconotus) punctatus*, wing length = 4.5 mm **8** *Lacconotus (Alcconotus) pinicola*, wing length = 5.1 mm. Photo credit: Darren Pollock, Eastern New Mexico University.

**Figures 9–10.**
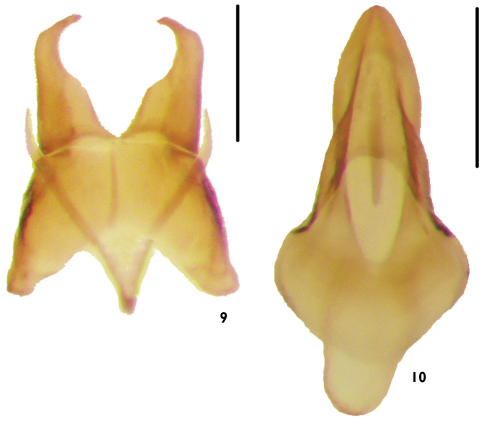
Male genitalia of *Lacconotus (Lacconotus) punctatus* **9** tegmen **10** median lobe. Scale bar = 0.25 mm. Photo credit: Darren Pollock, Eastern New Mexico University.

### Alcconotus

Pollock & Majka subgen. n.

urn:lsid:zoobank.org:act:7F51E3D0-3408-4469-BC83-EFD22E47750F

http://species-id.net/wiki/Alcconotus

#### Type species.

*Lacconotus pinicola* Horn, by present designation.

#### Derivation of name.

 a partial anagram of *Lacconotus*, in which its species was formerly placed.

#### Taxonomic notes.

Pollock (2002) stated that it might be necessary to establish a new genus for the two western species of *Lacconotus*, although no details were given to justify this possibility. There are many differences between the eastern and western species of *Lacconotus* (see description above, for *Lacconotus*), but the most significant reason for proposal of this new subgeneric name is the structure of the male sex patch, which differs greatly between *Lacconotus punctatus* and *Lacconotus pinicola*. It could be argued that this might justify separation into two genera; however, within the related genus *Mycterus* Clairville (Mycterinae) there are also significant differences in this structure. Also, there are significant differences in the structure of the bursa copulatrix and spermatheca between the two groups (see Figs 13–14); it is impossible to compare these intrageneric differences with other eurypine or mycterid taxa, since the internal female genitalia have yet to be studied in detail in most groups.

#### Description.

 See description above, for *Lacconotus*; characteristics unique to *Alcconotus* are indicated with the alternatives for *Lacconotus* (*s. str.*).

### Lacconotus (Alcconotus) pinicola

Horn comb. n.

http://species-id.net/wiki/Lacconotus_pinicola

Figs 2 –3 6 8 11–12 14 15 –16

Lacconotus pinicola Horn 1879: 338. – Type locality: Veta Pass [= La Veta Pass?], Colorado. LeConte 1879: 500, 506; Austin 1880: 41; Snow 1882: 44; Cockerell 1893: 334; Coquillett and Orcutt 1900: 54 [= *pallidus*?]; Wickham 1902: 297; Woodworth 1913: 194; Seidlitz 1917: 99; Leng 1920: 240; Blair 1928: 33; Van Dyke 1928: 257; Spilman 1951: 50, fig. 15; Hatch 1965: 88; Arnett 1983: 3; Campbell 1991: 267; Poole and Gentili 1996: 315; Pollock 2002: 532.
Lacconotus pallidus Van Dyke 1928: 256; Fall 1901: 32 (as *Lacconotus pinicola*), 177; Leng and Mutchler 1933: 36; Spilman 1951: 50; Crowson and de Viedma 1964; Pollock 2002: 531 (fig. 2.112), 532. syn. n.

#### Types.

(***Lacconotus pinicola***, all in MCZC).—lectotype (here designated), female, labeled: “Veta Pass 27.6 Col / 592 / [red] Type 7976 / [handwritten] Lacconotus pinicola (Schwz) / J.L. LeConte Collection / LECTOTYPE ♀ *Lacconotus pinicola* Horn 1879; design. D.A. Pollock 1994”. paralectotype. female, labeled: “Col / [blue] Para-Type 8047. / G.H. Horn Collection”, in MCZC.

#### Types.

 (***Lacconotus pallidus***, all in CAS).—holotype, male (CAS type # 2585), labeled “Mt. Wilson Cal. 6.13.3 / 7701 / Van Dyke Collection / Holotype [along left margin of label covered in red ink] ♂ Lacconotus pallidus Van Dyke". ALLOTYPE, female, labeled "Carmel, Monterey Co VI-4-1916 Cal. / Van Dyke Collection / Allotype [along left margin of label covered in red ink] ♀ Lacconotus pallidus Van Dyke". Four PARATYPES. Male, labeled "Carmel, Monterey Co VI-4-1916 Cal. / Van Dyke Collection / Paratype [along left margin of label] ♂ Lacconotus pallidus Van Dyke". Male, labeled "ParaisoSpgsCal V.28 1924 L.S. Slevin / L.S. Slevin Collection / Paratype [along left margin of label] Lacconotus pallidus Van Dyke". Female, labeled "ParaisoSprings V.31 1916 Cal. / L.S. Slevin Collection / Paratype [along left margin of label] Lacconotus pallidus Van Dyke". Female, labeled "Paraiso Springs V.29 1916 Cal. / CHAMISAL / L.S. Slevin Collection / Paratype [along left margin of label] Lacconotus pallidus Van Dyke".

#### Diagnosis.

*Lacconotus (Alcconotus) pinicola* may be distinguished from *Lacconotus punctatus* by the following features: body color ranging from testaceous to dark brown, uniform dorsally (Figs 2–3); antennae relatively long, subserrate; male sex patch on ventrite 2 small, oval, densely pubescent (Fig. 6); distribution in western North America (Fig. 15).

#### Re-description.

**Figures 11–12.**
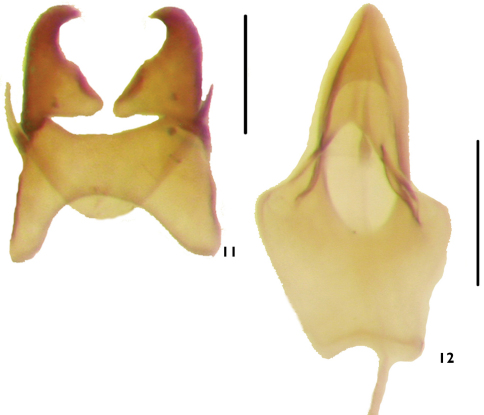
Male genitalia of *Lacconotus (Alcconotus) pinicola* **11** tegmen **12** median lobe. Scale bar = 0.25 mm. Photo credit: Darren Pollock, Eastern New Mexico University.

 (see Horn 1879 and Van Dyke 1928) – With general features of subgenus *Alcconotus* (as described above) with the following: TL 4.8–7.5 mm; GEW 1.5–2.3 mm; TL/GEW 2.8–3.4. Dorsal body surface uniformly testaceous to dark brown or piceous (Figs 2–3), without any color contrast; antennomeres 5–10 relatively elongate, subserrate; antennal sensilla not completely surrounding opening of antennomere, restricted to triangular side of antennomeres; wing membrane distinctly pigmented, veins very conspicuous (Fig. 8); male sex patch longitudinally oval, occupying about 2/3 length of ventrite 2, densely covered with short setae (Fig. 6), not bulbous or contrasting in color; tegmen of male genitalia (Fig. 11) short, parameres stout; bursa copulatrix (Fig. 14) very large, spherical.

**Figures 13–14.**
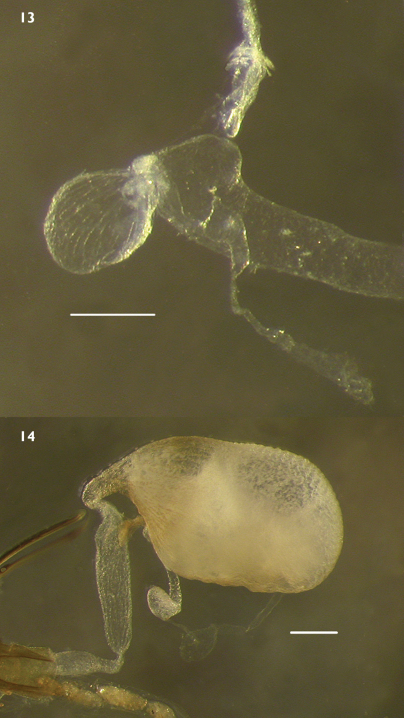
Internal female genitalia of *Lacconotus* **13** *Lacconotus (Lacconotus) punctatus* **14** *Lacconotus (Alcconotus) pinicola*. Scale bar = 0.25 mm. Photo credit: Darren Pollock, Eastern New Mexico University.

**Figure 15.**
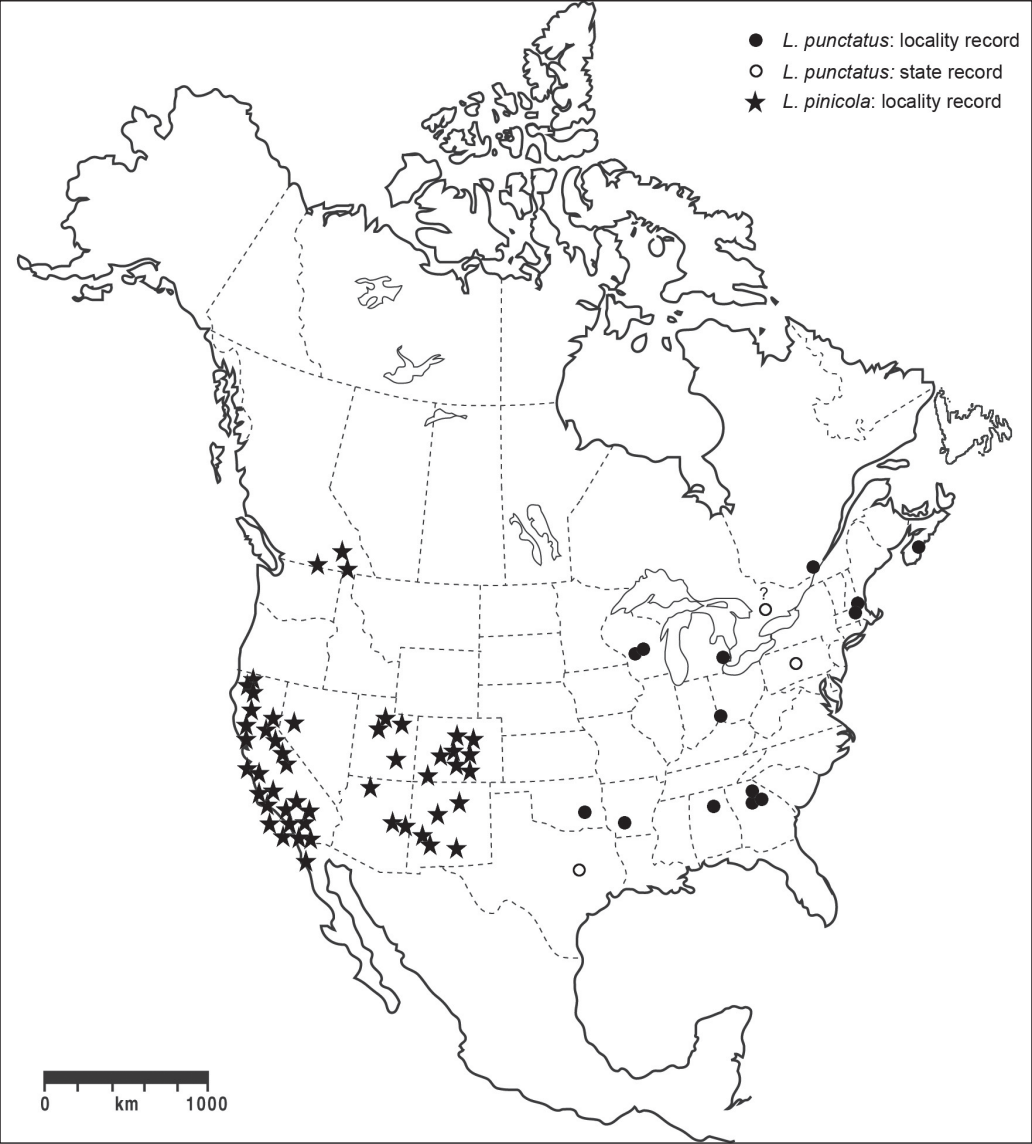
Distribution of *Lacconotus (Lacconotus) punctatus* and *Lacconotus (Alcconotus) pinicola* in North America and Mexico.

**Figure 16.**
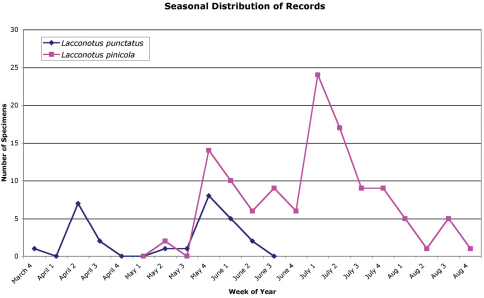
Phenology of *Lacconotus (Lacconotus) punctatus* and *Lacconotus (Alcconotus) pinicola* in North America and Mexico.

#### Notes.

Van Dyke (1928) established *Lacconotus pallidus* (as distinct from *Lacconotus pinicola*) based on its lighter color, relatively narrower pronotum, shorter relative length of the elytra, and deeper punctation. However, upon examination of the type series and other specimens, we have determined that the only feature of Van Dyke’s that withstands scrutiny is the habitus color. As well, more detailed examination has revealed that the male and female genitalia and male sex patch are virtually identical between *Lacconotus pinicola* and *Lacconotus pallidus*. One feature, mentioned by Van Dyke (1928) that does seem noteworthy is the somewhat restricted distribution of *Lacconotus pallidus* in southern California. We herein consider *Lacconotus pallidus* a pale “form” of *Lacconotus pinicola*.

*Lacconotus (Alcconotus) pinicola* is newly recorded from Arizona, Utah, and Baja California Norte in Mexico (see Appendix A). Published records of *Lacconotus pinicola* are from British Columbia (Hatch 1965), California (Fall 1901; Van Dyke 1928), Colorado (Horn 1879; Cockerell 1893; Van Dyke 1928), western Nevada (Horn 1879), and New Mexico (Snow 1882, 1906; Knaus 1907). The range of the species (Fig. 15) shows it to be widely distributed in the southwestern United States (Arizona, California, Colorado, Nevada, New Mexico, and Utah) extending south to Baja California Norte in Mexico, and in southeastern British Columbia. Specimens should be sought in intervening areas in Idaho, Oregon, and Washington to ascertain if these populations are actually disjunct.

A number of specimens examined were found on ponderosa pine (*Pinus ponderosa* Douglas ex. C. Lawson), including one specimen which was recorded as emerging from a dead *Pinus ponderosa* branch. It has also been found on Engelmann spruce (*Picea engelmanni* Parry ex Engelm.), scrub oak (*Quercus turbinella* Greene), and cherry (*Prunus* sp.). Specimens have been collected at UV lights, with malaise and flight-intercept traps, and by beating vegetation. Fall (1901: 177) wrote “...rare during May and June; found always on oaks, notwithstanding its name.” The larva of *Lacconotus pinicola* has been illustrated by Lawrence (1991), but not described in detail. The phenology information that is available (Fig. 16) indicates that adults can be found between 13 May and 29 August with two specimens having been found in the autumn (6 October and 6 November). The peak in adult numbers appears to be in the first half of July.

## Supplementary Material

XML Treatment for Lacconotus

XML Treatment for Lacconotus (Lacconotus) punctatus

XML Treatment for Alcconotus

XML Treatment for Lacconotus (Alcconotus) pinicola

## Appendix 1

### Non-type specimen records

***Lacconotus (Lacconotus) punctatus* LeConte**

**CANADA: NOVA SCOTIA: Lunenburg County:** Bridgewater, 16 June 2004, G. Selig, adjacent to mixed forest (NSMC, 1); **QUEBEC:** Montreal Island, 24 May 1902 (CUIC, 1).

**UNITED STATES: ALABAMA:** **Jefferson County:** Rocky Ridge, 17 April 1982 (AAAC, 1); Rocky Ridge, 31 March 1982, T. King, (AAAC, 1). **ARKANSAS: Hempstead County:** Hope Upland Wildlife Management Area (33°44'20"N; 93°38'59"W), 13 April 2003, J.P. Gruber, swept from low foliage in mixed forest/old field (1, WIRC); **MASSACHUSETTS: Middlesex County:** Tyngsboro, H.C. Fall collection, (MCZ, 1); state record only, (MCZ, 1). **MICHIGAN: Wayne County:** Detroit, Hubbard & Schwarz collection, (NMNH, 1). **NEW HAMPSHIRE: Strafford County:** 1 mi. SW Durham, 5 June 1981, 25 May 1982, 27 May 1988, 29 May 1988, 31 May 1988, 5 June 1990, water tower, W.J. Morse (DAPC, 7); 1 mi. SW Durham, 30 May 1982, 3 June 1982, W.J. Morse, water tower (MCZ, 2); 1 mi. SW Durham, 21 May 1982, D.S. Chandler, water tower (DAPC, 1). **OKLAHOMA: Latimer County:** April 1985, K. Stephan, beating dead oak (1, TAMU); Latimer County: May 2001, K. Stephan, ultra-violet light (1, TAMU). **PENNSYLVANIA:** state record only, H. Ulke collection, (CMNH, 1). **TEXAS:** state record only, H. Ulke collection, (CMNH, 1). **WISCONSIN: Monroe County:** Ft. McCoy, 1 mi. west of Big Rock, 12–15 June 1997, J.A. Maxwell, oak savanna, flight intercept trap (WIRC, 1); **Juneau County:** Necedah National Wildlife Refuge, 30 May 1996, 6 June 1996, K. Pope, flight intercept trap (WIRC, 4).

***Lacconotus (Alcconotus) pinicola* Horn**

**CANADA: BRITISH COLUMBIA:** Kaslo, 8 July, A.N. Caudell (NMNH, 1); Creston, 19 July 1946, G. Stace Smith (UBC, 1); Osoyoos, 2 July 1948, R. Scott, at light (UBC, 1).

**MEXICO: Baja California Norte.** Ensenada: S[ierr]a Juarez, 3.6 mi. SSE El Rayo, 2.vii.1960, E.L. Sleeper, (CASC, 1).

**UNITED STATES: ARIZONA:** **Apache or Navajo County:** McNary, 6 July 1945, F.H. Parker (UAIC, 1); **Apache County:** White Mts., 25 July 1944, Parker (UAIC, 1); **Coconino County:** 22 mi. S. Jacob Lake, De Motte Park cmpgrnd, 8700', 17 July 1969, L.N. & C.J. Bell, (CAS, 1). **CALIFORNIA:** [“*pallidus*" form] **Fresno or Tulare County:** Kings Canyon N[ational] P[ark], 24 June 1955, P.S. Bartholomew (CAS, 1); **Kern County:** Tehachapi Mts., Antelope Canyon, 18 July 1976, 6000 ft. (UCR, 2); **Los Angeles County:** Santa Catalina Island, Blackjack Rd. (33.3919°N, 118.4001°W), 17 June 2008, Caterino & Chatzimanolis (1, SBMN); Santa Catalina Island, nr. Echo Lk., (33.3974°N, 118.3946°W) 18 June 2008, Caterino & Chatzimanolis (1, SBMN); Angeles National Forest, Ruby Canyon (34.6060°N, 118.5523°W), 22 June 2007 (1, SBMN); Angeles National Forest, Big Dalton Canyon (34.1811°N, 117.7978°W), 13–23 June 2007, Caterino & Chatzimanolis, flight intercept trap (1, SBMN); Angeles National Forest, Tanbark Flat (34.2048°N, 117.7611°W), 23 June 2007, Caterino & Chatzimanolis, at light (1, SBMN); Muchmore, 29 July 1920, (MCZ, 1); Tanbark Flat, 30 June 1950, B. Adelson, at electric light (EMEC, 1); Tanbark Flat, 24 June 1950, H.M. Graham, (EMEC, 1); Santa Monica Mts., Fryman Canyon, 25 May 1991, J. Rifkind (WFBM, 1); county record only (NMNH, 1); Madera County: Bass Lake, 3 June 1942, *Pinus ponderosa* (TAMU, 1); S[ierra] Madre, June, (CAS, 1); Mt. Lowe, June (CAS, 2); **Madera County:** Bass Lake, 3 June 1942, *Pinus ponderosa* (TAMU, 1). **Marin County:** Phoenix Lake, 30 May 1927, H.H. Kelfer (CAS, 1); San Gabriel Mts., 5 June 1910, 3500 ft. (MCZ, 1); Mt. Wilson, 6 November 1904, 23 July 1905, 5 June 1917, (MCZ, 2; NMNH, 1); Mt. Wilson, 29 June 1940, G.P. Mackenzie, (UCRC, 1); Pasadena, 29 May 1897, 31 May 1897, June 1922, 22 June 1902, (MCZ, 5); Pasadena, 2 July 1926, (NMNH, 1); Pasadena, May, (CAS, 1; AAAC, 1); Pasadena, A. Fenyes, (CMNH, 3); Pasadena, (CUIC, 1; NMNH, 1; FMNH, 2); Pom[ona?] Mts., 6 October 1893, (MCZ, 1); Sequoia Nat. Park, Potwisha, 25 May 1929, 3000–5000 ft. (CAS, 1); Los Gatos, Hubbard & Schwarz, (NMNH, 1); Santa Cruz Mountains, July (FMNH, 2);Fairfax, 18 June 1939 (CAS, 1); **Monterey County:** UC Big Creek Reserve, Highlands Camp (36.062°N, 121.571°W), 31 May–8 June 2003, M. Caterino, flight-intercept trap (1, SBMN); Paraiso Springs, 31 May 1916, (CAS, 1); Paraiso Springs, 9 June 1932, L.S. Slevin (CAS, 1); **Napa County:** N. side Howell Mt., 2 mi. NNE Angwin, 5 June 1978, H.B. Leech, emerged ex dead branch of *Pinus ponderosa*, 1300 ft. (CAS, 1); **Orange County:** Silverado Cyn., 22 June 1958, E.L. Sleeper (CAS, 1); **Riverside County:** James Reserve (33.8081°N, 116.7784°W), 15 July 2006, Caterino & Chatzimanolis (1, SBMN); **San Bernardino County:** Oak Glen, 26 July 1967, M.J. Wargo (CDAE, 1); Gobbler's Knob (34.3116°N, 117.5835°W), 3 July 2005, M. Caterino (6, SBMN; 1 AAAC); Forest Home, 14 June 1928 (CAS, 1); [?Big] Bear Lake, 18 May 1919 (CAS, 1); **San Diego County:** Poway, F.E. Blaisdell (CAS, 1); **San Luis Obispo County:** Los Padres National Forest, Cuesta Ridge (35.3630°N, 120.6573°W), 9 July 2008, Caterino & Polihronakis (1, SBMN); **Santa Barbara County:** UC Sta. Cruz Isl. Res. (34.0013°N, 119.7512°W), 6 June 2005, M. Caterino & J. Jacobs, (SBMN, 1); UC Sta. Cruz Isl. Res. (34.0013°N, 119.7967°W), 6 June 2005, M. Caterino & J. Jacobs, (SBMN, 1); UC Sta. Cruz Isl. Res. (33.9963°N, 119.7281°W), 5–7 June 2005, Lindgren trap, M. Caterino & J. Jacobs, (SBMN, 1); UC Santa Cruz Island Res., Cañada del Puerto (34.0019°N, 119.7127°W), 13 May 2009, Caterino, Chatzimanolis, Hopp & Polihronakis, (SBMN, 1); UC Sedgwick Reserve (34.7246°N, 120.0351°W), 14 May 2005, M. Caterino, beaten from *Prunus*, (SBMN, 1); Los Padres National Forest, Fremont Tr. (34.5158°N, 119.8069°W), 19–27 June 2001, Malaise, E.I. Schlinger & S. Regan, (SBMN, 2);Freemont Tr. nr. Painted Cave, 8–15 July 2001, E. Schlinger & S. Regan, malaise trap (SBMN, 1); Arroyo Hondo Preserve, 25 mi. W of Santa Barbara (34.486°N, 120.135°W), 2 July 2003. M. Caterino (1, SBMN); Los Padres National Forest, 15 mi. NW of Santa Barbara, west of Camino Cielo (34°30'N, 119°49.8'W), 4 July 2001 M. Caterino, 900 m (1, SBMN); Los Padres National Forest, 5 mi. north of Santa Barbara (34°29.58'N, 119°41.13'W), 5 July 2001, M. Caterino, 1050 m (2, SBMN); 3 mi. N. Refugio Beach, 28 June 1965, J.R. Stephenson (CAS, 1); **Santa Clara County:** Los Gatos, Hubbard & Schwarz (NMNH, 1); Santa Cruz Mountains, July (FMNH, 2); **Santa Cruz County:** 4 mi. SE Big Basin, 4 July 1967, A.R. Gillogly, uv light (TAMU, 2); **Tulare County:** Sequoia Nat. Park, Potwisha, 25 May 1929, 3000–5000 ft. (CAS, 2); **Tuolumne County:** Phoenix Lake, 30 May 1927, H.H. Kelfer (CAS, 1); **CALIFORNIA:** [“*pinicola*" form] **Butte County:** 2.8 mi NW French Creek, 15 July 1990, light trap, W.D. Shepard & C.B. Barr, (CAS, 2). **El Dorado County:** Blodgett Forest, 18 mi. E. Georgetown, 1 July 1967, J. Powell, at light (EMEC, 1); Pollock Pines, 27 July-18 August 1987, R.B. Flint, blacklight (CSCA, 1); **Placer County:** Tahoe National Forest, Pineland Drive 3.2 km S. of Tahoe City, 1900 m, 30 July 1983, T.W. Davies (CAS, 1); **Siskiyou County:** 9 mi. NW Happy Camp, blacklight trap, 22 August 1982, F.D. Horn (CSCA, 1); **Trinity County:** Carrville, 10 June 1913, (CAS, 1); **Tuolumne County:** 4 mi. W. Pinecrest, 12 July 1961, J.G. Rozen (AMNH, 4); Lyons Dam Rd., 29 August 1968, W.F. & F.C. Tyson, attracted to black light (NMNH, 1); **COLORADO:** **Boulder County:** Longs P[ea]k Inn, 13 July 1926, E.C. Van Dyke, 9000 ft. (CAS, 1); Ward, July 1905, CKU (MCZ, 1); Custer County, T.D.A. Cockerell (NMNH, 1); **Delta County:** Paonia, 14 June 1926, E.C. Van Dyke (CAS, 1); **El Paso County:** Waldo Canyon, 25 June 1916, W.D. Edmonston, *Pinus ponderosa* (NMNH, 1); Manitou [?Springs], 23 June 1926, E.C. Van Dyke (CAS, 1); **Grand or Jackson County:** Rabbit Ears [Pass], 24 July 1930, *P*[*icea*] *engelmanni* (NMNH, 1); **Huerfano County:** [La] Veta Pass, 21 June, Hubbard & Schwarz (NMNH, 1); [La] Veta Pass, 1 July, F.C. Bowditch Coll. (MCZ, 1); **La Plata County:** Durango, 10 July 1968, E.C. Becker, beating scrub oak, 7000' (CNC, 5); Durango, 7 July 1968, E.C. Becker, 7500' (CNC, 3); Durango, Horse Canyon, 21 July 1968, E.C. Becker, beating scrub oak, 7000' (CNC, 1); **Teller County:** Florissant, 7 July 1902, S.A. Rohwer (MCZ, 1); County unknown: Florentine, July 1879, Pourtales (MCZ, 1); county unknown, Pike Nat. For, Top-of-the-World Camp, 7 August 1976, B.F. & J.L. Carr (CNC, 1); State record only, Ulke Collection (CMNH, 1; MCZ, 1). **NEW MEXICO:** **Bernalillo County:** Albuquerque, 29 May 1994, 10 June 2007, R. Buss, UV light (2, RBC); **Socorro County:** Bear Trap Camp, 28 mi. SW Magdalena, 7 July 1965, F. P. & M. Rindge, 8500' (AMNH, 1). **Lincoln County:** Cedar Creek, 5 miles N. Ruidoso, 2 July 1961, F., P. & J. Rindge, 7500' (AMNH, 1); Holman Pass, NW of Holman, C.C. Hoff, (AMNH, 1); State records only, Ulke Collection, (CMNH, 3); F.A. Eddy Collection (MCZ, 2). **UTAH: Garfield County:** Lonesome Beaver, Henry Mts., 25 July 1968, A.T. Howden, 7500' (CNC, 1); **Salt Lake County:** 6.4 km E. Granite, R.F. Rockwell, Wasatch-Cache National Forest Survey (40°34'20"N; 111°43'47"W), 21 July 1998, 29 July 1998, 5 August 1998, 22 August 1998, 1945m, uv light trap (CMNH, 12); **Utah County:** Squaw Peak near summit, 5.3 km SSE Springdell, (40°16'57"N; 111°36'19"W), 16 July 1998, R.F. Rockwell (Uinta National Forest Survey), grassy knoll, 2390 m, uv light trap (CMNH, 1); American Fork Canyon, near mouth, 8.0 km N. Pleasant Grove (40°26'08"N; 111°43'45"W), 12 August 1998, R.F. Rockwell (Uinta National Forest Survey), 1580 m, uv light trap (CMNH, 1).
